# Evidence of co-exposure with *Brucella* spp, *Coxiella burnetii*, and Rift Valley fever virus among various species of wildlife in Kenya

**DOI:** 10.1371/journal.pntd.0010596

**Published:** 2022-08-08

**Authors:** Francis Gakuya, James Akoko, Lillian Wambua, Richard Nyamota, Bernard Ronoh, Isaac Lekolool, Athman Mwatondo, Mathew Muturi, Collins Ouma, Daniel Nthiwa, Earl Middlebrook, Jeanne Fair, John Gachohi, Kariuki Njenga, Bernard Bett

**Affiliations:** 1 Wildlife Research and Training Institute, Naivasha, Kenya; 2 International Livestock Research Institute, Nairobi, Kenya; 3 Department of Biomedical Science, Maseno University, Kisumu, Kenya; 4 Kenya Wildlife Service, Nairobi, Kenya; 5 Zoonotic Disease Unit, Nairobi, Kenya; 6 Department of Medical Microbiology and Immunology, Faculty of Health, University of Nairobi, Kenya; 7 Faculty of Veterinary Medicine, Freie Universität Berlin, Germany; 8 Department of Biological Sciences, University of Embu, Embu, Kenya; 9 Los Alamos National Laboratory, New Mexico, United States of America; 10 Washington State University, Global Health Programme, Nairobi, Kenya; 11 School of Public Health, Jomo Kenyatta University of Agriculture and Technology, Nairobi, Kenya; Instituto Butantan, BRAZIL

## Abstract

**Background:**

Co-infection, especially with pathogens of dissimilar genetic makeup, may result in a more devastating impact on the host. Investigations on co-infection with neglected zoonotic pathogens in wildlife are necessary to inform appropriate prevention and control strategies to reduce disease burden in wildlife and the potential transmission of these pathogens between wildlife, livestock and humans. This study assessed co-exposure of various Kenyan wildflife species with *Brucella spp*, *Coxiella burnetii* and Rift Valley fever virus (RVFV).

**Methodology:**

A total of 363 sera from 16 different wildlife species, most of them (92.6%) herbivores, were analysed by Enzyme-linked immunosorbent assay (ELISA) for IgG antibodies against *Brucella* spp, *C*. *burnetii* and RVFV. Further, 280 of these were tested by PCR to identify *Brucella* species.

**Results:**

Of the 16 wildlife species tested, 15 (93.8%) were seropositive for at least one of the pathogens. Mean seropositivities were 18.9% (95% CI: 15.0–23.3) for RVFV, 13.7% (95% CI: 10.3–17.7) for *Brucella spp* and 9.1% (95% CI: 6.3–12.5) for *C*. *burnetii*. Buffaloes (n = 269) had higher seropositivity for *Brucella* spp. (17.1%, 95% CI: 13.0–21.7%) and RVFV (23.4%, 95% CI: 18.6–28.6%), while giraffes (n = 36) had the highest seropositivity for *C*. *burnetii* (44.4%, 95% CI: 27.9–61.9%). Importantly, 23 of the 93 (24.7%) animals positive for at least one pathogen were co-exposed, with 25.4% (18/71) of the positive buffaloes positive for brucellosis and RVFV. On molecular analysis, *Brucella* DNA was detected in 46 (19.5%, CI: 14.9–24.7) samples, with 4 (8.6%, 95% CI: 2.2–15.8) being identified as *B*. *melitensis*. The Fisher’s Exact test indicated that seropositivity varied significantly within the different animal families, with *Brucella* (p = 0.013), *C*. *burnetii* (p = <0.001) and RVFV (p = 0.007). Location was also significantly associated (p = <0.001) with *Brucella spp*. and C. *burnetii* seropositivities.

**Conclusion:**

Of ~20% of Kenyan wildlife that are seropositive for *Brucella* spp, *C*. *burnetii* and RVFV, almost 25% indicate co-infections with the three pathogens, particularly with *Brucella spp* and RVFV.

## Author summary

Infection of an animal with more than one pathogen may result into a more devastating impact. Only few studies have investigated co-infection with multiple pathogens in wildlife, despite their key role as reservoirs of zoonotic diseases. Therefore, there is need for investigations on co-infection with neglected zoonotic pathogens in wildlife to inform prevention and control approaches and reduce disease impact in wildlife and potential transmission of these pathogens between wildlife, livestock, and humans. This study assessed co-exposure of various Kenyan wildlife species with three zoonotic pathogens, including *Brucella spp*, *Coxiella burnetii* and Rift Valley fever virus (RVFV). Results from this study revealed widespread, but varied exposure levels to the three individual pathogens within the several wildlife species. Likewise, the study also found the presence of co-exposure with the three pathogens. The findings from this study points to the need for establishment of surveillance and control programmes that target multiple pathogens in the wildlife populations to reduce the risk of transmission of infectious pathogens in wildlife and their zoonotic transmission.

## Introduction

Zoonotic infections remain a key global threat, with emergence of new zoonoses such as Marburg and Ebola, as well as the resurgence and persistence of existing zoonotic infections, leading to devastating social, economic and health outcomes [1,2]. Human-animal interactions underpin the transmission of zoonoses, with the zoonotic pathogens being mobilized between vertebrate animals (both domestic and wildlife) and humans through various routes including direct contact with infected hosts, or indirect contact via the food chain, the environment or intermediate vectors such as ticks and mosquitoes [3]. Wildlife have been implicated in the emergence, maintenance and spillage of over 70% of zoonotic diseases [4–7]. Multisectoral, One Health strategies are thus recommended to predict, mitigate and control zoonotic infections [2].

Although many zoonotic diseases have a global distribution, several zoonoses categorized as “neglected zoonotic diseases” (NZDs), are associated with poverty, and huge public health and economic burden amongst the global poor [8]. Developing countries in Africa and Asia have higher disease burden of these NZDs such as brucellosis, Q fever and Rift Valley fever, which are also classified as extremely dangerous pathogens (EDPs) [9–11]. The prevalence of these EDPs is usually underestimated and the risk levels remain understudied. Hence their control and prioritization in surveillance programmes in most developing countries remains underfunded [1,8]. Thus the first step towards understanding these zoonotic pathogens in a One Health framework is to understand their prevalence in wildlife populations.

Brucellosis, Q fever and RVF are associated with acute undifferentiated febrile illness in humans, which may progress to chronic disease with systemic manifestations [12–15]. In livestock, these three diseases cause abortion storms, stillbirths, premature and/or weak offspring, with significant reproductive and economic losses to farmers [13,16,17]. Humans acquire the causative pathogens through direct contact with tissues or body fluids of infected animals e.g. aborted materials or carcasses, aerosols in the case of Q fever and consumption of unpasteurized milk and dairy products in the case of brucellosis.

Increasing reports point to the role of wildlife as potential reservoirs for *Brucella* spp, *Coxiella burnetti* and RVF virus, although systematic studies on the ecology and epidemiology of these pathogens in wild animals remains limited. Recently, Simpson and co-workers [18], reported brucellosis in many species of wildlife in Africa, with buffalo, being implicated as maintenance hosts for *Brucella* species. Although *C*. *burnetii* is known to infect wildlife species [19], this pathogen was not detected in a previous survey that screened variety of wildlife species in Kenya [20]. In the case of RVF virus, little is known on the role of wildlife, although some studies suggest that wild animals may constitute a reservoir system for RVF virus during inter-epidemic seasons and in enzootic areas [21,22].

While several studies have shown exposure and potential circulation of *Brucella spp*, *Coxiella burnetti* and RVF virus in various wildlife species, the evidence for co-exposure of multiple species of these pathogens within wildlife populations, remains unexplored. Simultaneous spillover of multiple pathogens from wildlife to livestock and humans are likely to have implications on disease pathogenesis, disease course and health outcomes [23]. Co-infections may alter virulence of pathogens and subsequent disease outcomes in the hosts [23–25]. Across a wide range of pathogen taxa, co-infections generally lead to worse health outcomes for hosts and increase within host pathogen titers, altering transmission ecologies. Understanding these dynamics is therefore useful not only in furthering our knowledge of wildlife in the epidemiology of Q fever, RVF, and brucellosis, but also in calibrating existing risk models for these diseases in various contexts. Hence, the current study analyzed the seropositivity of Q fever, RVF, and brucellosis in 16 wildlife species belonging to the orders Artiodactyla, Perissodactyla and Carnivora from various parts of Kenya. We performed a retrospective study where samples collected during routine animal immobilizations for interventions due to traumatic injuries, translocation and collaring were tested. The findings give preliminary information on occurrence of co-infections in wildlife by geographic region, which has not been previously reported. We examined serological evidence of exposure to single and multiple pathogens, as well as molecular analysis of *Brucella* spp. in the different wildlife species.

## Materials and methods

### Study area and samples

**Fig 1:**
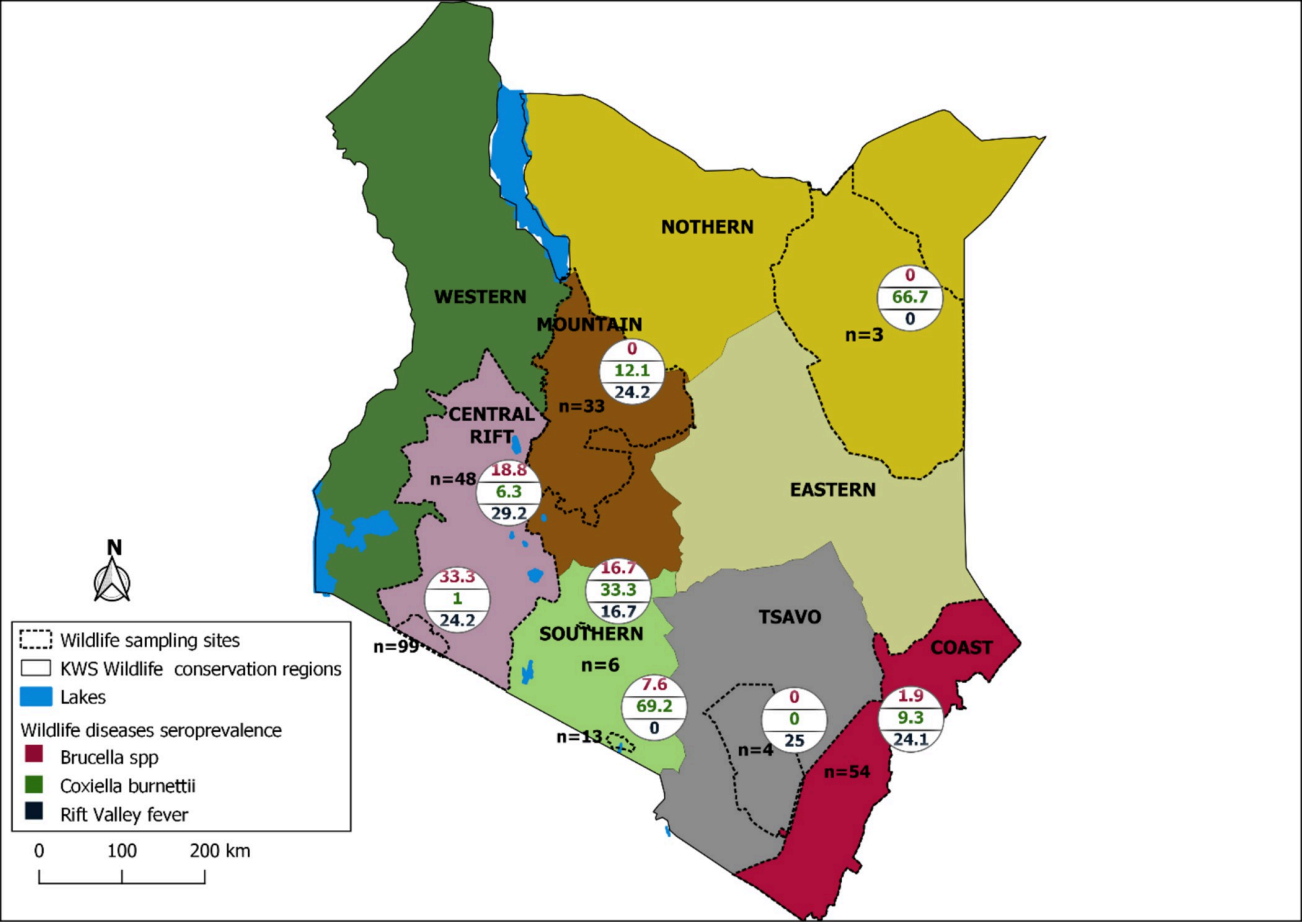
Total number of tested animals categorized by region sampled and corresponding positivity rates for antibodies against *Brucella*, *C. burnetii* and RVFV. The country boundary was obtained from https://gadm.org/download_country.html. The wildlife parks region boundaries are author-generated based on spatial boundaries provided by the Kenya Wildlife Service (KWS). The base layers used were appropriately licensed (https://gadm.org/license.html).

A total of 363 archived sera samples from 16 wildlife species (Artiodactyla, Perissodactyla and Carnivora) were obtained from the Kenya Wildlife Service (KWS) in Nairobi. These samples were collected during routine veterinary interventions and disease surveillance activities conducted by the KWS between 2010 and 2021, and stored at -20°C. The samples originated from eight wildlife areas in Kenya including Central Rift area, Maasai Mara ecosystem, Coastal area, Tsavo ecosystem, Laikipia-Samburu ecosystem, Amboseli ecosystem, Nairobi National park, and surrounding areas and Wajir area (Fig 1).

### Serology

The serological testing of sera samples to detect the presence of antibodies against *Brucella*, *Coxiella burnetii* and RVFV was done using Enzyme-linked immunosorbent assays (ELISA), at the International Livestock Research Institute (ILRI), in Nairobi, Kenya. Individual serum samples were screened in duplicates for antibodies against *Coxiella burnetii*, RVFV and *Brucella*. The RVFV ELISA assay was done using ID screen Rift Valley Fever Competition Multispecies ELISA kit (IDvet innovative diagnostics, France) whereas ID screen Brucellosis Serum Indirect Multispecies ELISA kit (IDvet innovative diagnostics, France) was used for *Brucella*, and PrioCHECK Ruminant Q Fever AB Plate ELISA Kit (Applied Biosystems, Thermo Fisher Scientific) for *C*. *burnetii*. All the assays were done as per the manufacturer’s instructions and the optical densities were read at wave lengths specified for each kit using BioTek ELISA reader (Synergy HT, BioTek Winooski, VT 05404 United States).

### Molecular analysis using Real time PCR to detect *Brucella* species

Further molecular analysis were done to identify the circulating *Brucella* species among animals. Total DNA was extracted from 280 serum samples using QIAamp DNA Blood Mini Kit following the manufacturer’s instructions except for elution which was done using 70μl of buffer AE. The purity and quantity of eluted DNA was determined using NanoDrop 1000 UV-Vis spectrophotometer (Thermo Scientific, Waltham, MA, USA) and stored at -20°C. *Brucella* genus was determined by Real-time PCR targeting *Bcsp31* using the primers and probes listed in S1 Table. The samples whose amplification threshold value was less than 40 were classified as PCR positive for *Brucella*. Multiplex Real time PCR was done on PCR-positive samples using species-specific primers and probes previously developed for *B*. *melitensis*, *B*. *abortus* and *B*. *suis* (S1 Table). Besides the test samples, each assay included a non-template (negative control), and a positive control DNA for each *Brucella* species.

The PCR amplifications were done using 2x Luna Universal Probe qPCR master mix (New England BioLabs, MA, USA), in a 20 μl reaction volume as previously described [26]. Amplification was performed on QuantStudio 5 Real-time PCR System (Life Technologies Inc.) with the following conditions: Initial DNA denaturation at 95°C for 1 min, followed by 42 cycles of denaturation at 95°C for 15 secs and 1 min of annealing/amplification at 57°C.

### Statistical analysis

Data were recorded and cleaned in Microsoft Excel version 2018 (IBM, California). All statistical analysis were performed in R statistical software version 3.6.3 [27]. The packages *DescTools* and *gmodels* within R, were used to perform all descriptive analysis including the estimation of positivity rates and 95% confidence intervals. The independent factors assessed for their association with the animal-level seropositivity status of the three-targeted pathogens included; age category, sex, location and animal species were determined using the Fisher’s Exact test. However, some of the data had incomplete information on categorical factors; 71.1% of the records had information on animal species and location, while 58.1% and 48.8% had information on animal sex and age, respectively. The number of records varied by species. The varied numbers of the responses recorded for the different variables limited the use of multivariable analysis. Seropositivites were therefore calculated for only those species that had at least 30 samples, with exception for the animal categories. Tsavo ecosystem and Wajir area were excluded from the risk factor analysis due to low numbers of animals from these two regions.

### Ethical statement

The samples were collected by KWS during their routine surveillance and animal translocation activities and as such no ethical approval was required for the work.

## Results

### Wildlife population summary and Elisa esults

A total of 363 sera samples, comprising 199 samples from buffaloes (*Syncerus caffer*), 36 giraffes (*Giraffa camelopardalis*), 21 zebras (*Equus burchelli*), 17 elands (*Taurotragus*), 15 oryxes (*Oryx beisa*), 11 waterbucks (*Kobus ellipsiprymnus*), 11 gazelles (*Gazella spp*), 9 impalas (*Aepyceros melampus*), 8 cheetahs (*Acinonyx jubatus*), 8 elephants (*Loxodonta africana*), 8 warthog (*Phacochoerus aethiopicus*), 7 rhinos (*Dicerosbicornis*), 5 lions (*Panthera leo*), 4 wildebeests (*Connochaetes taurinus*), 3 hartebeest (*Alcelaphus buselaphus coxii*), and 1 leopard (*Panthera pardus*) were analyzed. The samples were sourced from Maasai Mara ecosystem (n = 100), Coastal area (n = 54), Central Rift area (n = 48), Laikipia-Samburu ecosystem (n = 32), Southern ecosystem (n = 10) and Wajir area (n = 3). The rest of the samples (n = 103) did not have information on their source. Of all the 211 samples with information on animal sex, 119 (56.4%) were males while 92 (43.6%) were females.

Amongst the three tested pathogens, RVFV had the highest seropositivity of 18.9% (95% CI: 15.0–23.3), followed by *Brucella* 13.7% (95% CI: 10.3–17.7) and *Coxiella burnetii* 9.1% (95% CI: 6.3–12.5) (Table 1). All except one (15/16) of the wildlife species had detectable antibodies against one or more of the three pathogens (Table 1), with only hartebeest (n = 3) testing negative for all the pathogens.

**Table 1:** Summary of the number of wildlife species tested and proportions of seropositive animals for *Brucella*, *C*. *burnetii* and RVFV.

Animals				*Brucella*		*C*. *burnetii*		RVFV	
Categories	Families	Species	Total no. Tested	No. positive	% positive (95% CI)	Number Positive	% positive (95% CI	Number of positive	% positive (95% CI)
Herbivores	*Bovidae*	Buffaloes	199	44	22.1 (16.5–28.)	5	2.5 (0.8–5.7)	41	20.6 (15.2–26.9)
		Eland	17	2		1		8	
		Wildebeest	4	0		0		1	
		Hartebeest	3	0		0		0	
		Gazelle	11	0		1		0	
		Impala	9	0		1		3	
		Waterbuck	11	0		3		0	
		Oryx	15	0		0		10	
		Total	269	46		11	4.1 (2.2–6.4)	63	23.4 (18.6–28.6)
	*Giraffidae*	Giraffe	36	1		16	44.4 (27.9–61.9)	2	5.6 (0.7–18.70
	*Suidae*	Warthog	8	1		0		0	
	*Elephantidae*	Elephant	8	0		0		2	
	*Equidae*	Zebra	21	0		3		0	
	*Rhiocerotidae*	Rhino	7	0		3		1	
	Total		349	48	13.7 (10.3–17.2)	33	9.4 (6.5–12.3)	68	19.4 (15.5–23.6)
Carnivores	*Felidae*	Leopard	1	1		0		0	
		Lion	5	1		0		0	
		Cheetah	8	0		0		1	
		Total	14	2	14.3 (7.1–35.2)*	0	0.0	1	7.1 (0.0–19.1)*
Overall			363	50	**13.7** (10.3–17.7)	33	**9.1** (6.3–12.5)	69	**18.9 (**15.0–23.3)

Key:

* Low numbers of animals. Therefore, positivity estimates should be treated with caution

### Factors associated with the seropositivity of *Brucella*

Positivity rates differed significantly between locations for *Brucella* (Fisher’s Exact Test, P = < 0.001). A relatively higher proportion was recorded among animals sampled in Maasai Mara ecosystem 33.4% (33/97), Central Rift area 19.1% (9/47), and none from both Laikipia-Samburu ecosystem (0/29) and Coastal area (0/34) (Table 2 and Fig 1). Exposure levels for *Brucella* also differed significantly by animal families (Fisher’s Exact Test,p = < 0.013), with antibodies against *Brucella* being detected more in *Bovidae* 17.1% (46/269) than Giraffidae 2.8% (1/36). No association was also observed between *Brucella* positivity with sex (Fisher’s Exact Test, p = 0.864), and age category (Fisher’s Exact Test, p = 0.571).

**Table 2:** Factors associated with Brucella, C.burnetii and RVFV positivity.

Factors		*Brucella*		*C*. *burnetii*		RVFV	
Variable	Category	Positivity	p-value	Positivity	p-value	positivity	p-value
Sex	Male	5% (5/100)	0.864	16% (16/100)	0.021	15% (15/1100)	0.792
	Female	7.9% (6/76)		5.3% (4/76)		18.4% (14/76)	
Families	*Bovidae*	17.1% (46/269)	0.013	4.1% (11/269)	<0.001	23.4% (63/269)	0.007
	*Giraffidae*	2.8% (1/36)		44.4% (16/36)		5.6% (2/36)	
Age category	Sub-adult	0.0% (0/5)		0.0% (0/5)		0.0% (0/5)	
	Adult	5.7% (5/87)	0.751	14.9% (13/87)	0.458	8.0% (7/87)	0.667
Location	Southern ecosystem	0.0% (0/11)	<0.001	81.8% (9/11)	<0.001	0.0% (0/11)	0.130
	Central Rift area	19.1% (9/47)		6.4% (3/47)		29.8% (14/47)	
	Coastal area	0.0% (0/34)		5.9% (2/34)		38.2% (13/34)	
	Laikipia-Samburu ecosystem	0.0% (0/29)		10.3% (3/29)		27.6% (8/29)	
	Maasai Mara ecosystem	34.0% (33/97)		1.0% (1/97)		24.7% (24/97)	

### Factors associated with the seropositivity of *C*. *burnetii*

Positivity rates differed significantly between locations for *C*. *burnetii* (Fisher’s Exact Test, p = <0.001). The Southern ecosystem had the highest positivity rate of 81.8% (9/11), Laikipia-Samburu ecosystem 10.3% (3/29), Central Rift 6.4% (3/47), Coastal area 5.9% (2/34), and Maasai Mara 1.0% (1/97). There was also significant differences in the positivity rates observed between the various animal families (Fisher’s Exact Test, p <0.001). *Coxiella burnetii* antibodies were detected in *Giraffidae* 44.4% (16/36), and *Bovidae* 4.1% (11/269). Likewise, a higher proportion of positivity was observed in males 16% (16/100) compared to females 5.3% (4/76), (Fisher’s Exact Test, p = 0.021). However,there was no association observed between the seropositivity of *C*.*burnetii* and age category (Fisher’s exact test, p = 0.458).

### Factors associated with seropositivity of RVF

The positivity status differed by animal families (Fisher’s Exact Test, p = 0.007). *Bovidae* 23.4% (63/269), and *Giraffidae* 5.6% (2/36). No statistical association was found between RVF positivity with location (Fisher’s Exact Test, p = 0.130), animal sex (Fisher’s Exact Test, p = 0.792) and age category (Fisher’s Exact Test, p = 0.667).

### *Brucella*, *C*. *burnetii and RVFV* co-exposure in wildlife species

Evidence of co- exposure with antibodies against *Brucella*, *C*. *burnetii* and RVFV was observed in different wildlife species. Of 93 animals positive for at least one pathogen, 23 (24.7%) were co-exposed, with 25.4% (18/71) of the positive buffaloes positive for brucellosis and RVF. The overall co-exposure with antibodies from any of the three pathogens was 24.7% (95% CI: 16.4–34.8). A relatively higher co-exposure was detected between *Brucella* and RVFV 22.7% (95% CI: 14.4–32.9), followed by *C*. *burnetii* and RVFV14.3% (95% CI: 1.7–42.8). Notably, there was no co-exposure between *Brucella* and *C*. *burnetii* antibodies for any species, whereas RVFV had co-exposure with either *Brucella* and *C*. *burnetii* (n = 1) (Table 3).

**Table 3:** Showing proportion of co-exposure with *Brucella*, *C*. *burnetii* and RVFV antibodies in different wildlife species. Only wildlife species that had an exposure with at least one of the pathogens were included in the analysis for co-exposure.

Wildlife species		*C*. *burnetii* and RVF virus		*Brucella* and RVF virus		*C*. *burnetii* and *Brucella*	Co-exposure with any of the three pathogens	
	Total N	N	% positive (95% CI)	Total n	% positive (95% CI)	Total n	Total n	% positive (95% CI)
Buffaloes	71	0	0.0	18	25.4 (15.7–37.1)	0	18	25.3 (15.8–37.1)
Eland	9	1	11.1(0.3–48.2)	1	11.1 (0.3–48.2)	0	2	22.2 (2.8–60.0)
Giraffe	8	0	0.0	1	12.5 (0.3–52.6)	0	1	12.5 (0.3–52.6)
Rhino	2	1	50 (1.3–98.7)	0	0.0	0	1	50.0 (1.3–98.7)
Impala	3	1	33.3 (0.8–90.6)	0	0.0	0	1	33.3 (0.8–90.6)
Total	93	3	14.3 (1.7–42.8)	20	22.7 (14.4–32.9)	0	23	24.7 (16.4–34.8)

Key: N total number of animals that tested positive for any of the three pathogens (both single and co- exposure), n number of co-exposure cases, CI Confidence Interval.

### Molecular analysis for detection of *Brucella* spp

*Brucella* DNA was detected in eight out of all the 16 analyzed wildlife species. An aggregate of 46 samples were positive for *Brucella* species DNA by PCR (Table 4). A total of four samples (3 buffaloes and 1 giraffe) tested positive for *B*. *melitensis*, while none of the samples amplified with *B*. *suis* and *B*. *abortus* species targets (S1 Fig).

**Table 4:** Showing wildlife species with the genus *Brucella* PCR positive results. The total and distribution of samples positive for genus *Brucella*, *B*. *abortus*, *B*. *melitensis* and *B*. *suis*.

Wildlife species		Total number of positive samples			
Species	Total tested	Genus *Brucella*.	*B*. *abortus*	*B*. *melitensis*	*B*. *suis*
**Buffaloes**	177	32	0	3	0
**Cheetah**	6	1	0	0	0
**Eland**	9	3	0	0	0
**Elephant**	5	2	0	0	0
**Giraffe**	20	5	0	1	0
**Lion**	4	1	0	0	0
**Oryx**	6	1	0	0	0
**Warthog**	8	1	0	0	0
**Total**	235	46 (19.5%)	0 (0.0%)	4 (8.6%)	0 (0.0%)

## Discussion

This study moved from the widely-used approach of investigating single pathogens to a simultaneous investigation of multiple pathogens, each with a different transmission mode, to fill the existing knowledge gap on the co-exposure to one viral (RVFV) and two bacterial (*Brucella and C*. *burnetii*) pathogens in wildlife populations in Kenya. Co-infection is widely recognized as one of the leading drivers of pathogen evolution and epidemiology [23,28], with more devastating epidemics observed in co-infected populations. We present evidence of co-exposure to *Brucella*, *C*. *burnetii* and RVFV exposure in various wildlife species in Kenya. We also observed variations in positivity rates of each of the three pathogens as a single exposure. However, co-occurrence between *Brucella* and *C*. *burnetii* antibodies were not detected in our data. We also found evidence of *Brucella* DNA in several wildlife species and *B*. *melitensis* DNA in buffaloes and giraffes.

We found an overall co-occurrence of *Brucella*, *C*. *burnetii* and RVFV antibodies in wildlife to be 24.7%, which is higher than the recently reported 15.0% of co-occurrence of the same pathogens in camels in Kenya [29]. Interestingly, we observed co-exposures with RVFV as common, more frequently with *Brucella* and less frequently with *C*. *burnetii*. These findings suggests that wildlife species could play a significant role in maintaining and transmitting multiple zoonotic pathogens at the same time [4,7]. Indeed wildlife species are known to be reservoirs of many zoonotic pathogens, which may spillover to either livestock and humans directly, or via an appropriate intermediate host [30]. Further, the increasing human population, coupled with shrinking and degraded wildlife habitats, are increasing wildlife population densities and could be responsible for transmitting infectious diseases and the relatively higher rate of co-occurrence of pathogens [31].

Our study found no evidence of co-exposure to *Brucella* and *C*. *burnetii*, despite their single occurrence in the same wildlife populations. Nevertheless, this coinfection was reported in domestic animals in Guinea [32] and therefore, we cannot entirely rule out the possibility of co-exposure to the two pathogens in wild species. Further, our ongoing unpublished studies have also identified *Brucella* and *C*. *burnetii* co-exposure in livestock belonging to the *Bovidae* family, to which some wildlife species investigated here belong. Nevertheless, these findings highlight the need to fully investigate the potential microbial interactions amongst *Brucella* spp. and *C*. *burnetii* in wildlife hosts.

We observed a higher rate of seropositivity to *Brucella* species (33%) and RVFV (20.4%) in the Maasai Mara ecosystem relatively to the rest of the areas in this study, suggesting an ecology permissive for transmission of both pathogens. *Brucella* species can survive in the environment for several months in cool and moist conditions which are also necessary for cryptic RVFV vectors breeding [33]. In addition, scavenging of contaminated placentas and weak offspring by predators, could be an additional risk factor for environmental transmission through mechanical dispersal for this pathogen. Other studies have shown the positivity rate of *Brucella* was similar for cattle (31.8%) and human (40.8%) in the same region, where there are intense wildlife and livestock interaction [26,34]. This highlights the interaction between wildlife, livestock, and humans in *Brucella* disease ecology. High wildlife and livestock population densities, climatic and land-use changes, and the perennial human-wildlife conflict in this ecosystem all lead to increasing wildlife-livestock-human interaction, making it a disease transmission hotspot posing public health risks [35]. This and other similar ecosystems could be ideal for investigating risk factors associated with zoonosis co-occurrence/co-infection dynamics. Although close contact between wildlife, human and livestock enable transmission of zoonotic pathogens [34], the directionality of transmission, could not be deduced from the current study; are these wildlife acting as reservoir hosts, or are they transmission dead ends. Therefore, further research involving a multidisciplinary, One Health approach is needed to fully explore the complex transmission dynamics of zoonotic pathogens that we studied and others in human-wildlife-livestock interfaces to inform integrated program for concurrent control of zoonoses.

The exposure levels for both *Brucella spp* and *C*. *burnetii* differed significantly, depending on the study locations, corroborating with reports of clustering of *Brucella spp*, *C*. *burnetii* and cases of other infectious diseases in domesticated animals [26,34]. However, a statistical association between location and animal species with exposure levels to RVFV antibodies was not found, contrary to previous reports [36,37]. Thus, the observed differences in exposure levels in different locations perhaps arose from spatial differences in environmental factors and the animal species present in various locations, considering that positivity rates also differed significantly in the different animal species. Future studies should target specific host species with unbiased sampling to better identify driving factors of co-exposure or co-infection. Regardless of the limitations of using archived samples in the current study, these findings have implications on the spread of zoonotic disease through wildlife movement, e.g., through natural migrations and wildlife translocations. Policies need to integrate disease risk analyses during wildlife translocations to prevent the introduction of pathogens to new areas or to spread to other wildlife, humans, and livestock in the destinations.

By highlighting evidence of exposure to three zoonotic pathogens, this study draws attention to potential anthropogenic activities that may result in spillovers of brucellosis, RVF, and Q fever infections from wildlife to humans and livestock. Emergence and spread of zoonotic infections is driven by several anthropogenic factors, including habitat fragmentation and degradation, increasing consumption of animal-derived foods; agricultural intensification; human encroachment in wildlife habitats and climate change, which cumulatively increase human-animal interactions. The hunting and trade of wildlife for meat may pose a heightened threat for potential exposure of humans to *Brucella*, *C*. *burnetii* and *RVFV*, for which slaughter, handling, and consumption of contaminated carcasses are major risk factors [35,38–39]. Our findings that wild ungulates (especially buffalo) had the highest exposure rates for all the pathogens, coupled with the fact that wild ungulate species are predominantly hunted for bushmeat in eastern Africa [36,40–43], underpin the potential risk of these and other anthropogenic activities in wildlife zoonotic transmission. Additional studies geared specifically to investigate these pathogens from a One Health perspective are warranted. These will target the interfaces between wildlife, livestock, and humans for instance, testing for infection with multiple pathogens where water and land resources are shared.

The results from molecular analysis of *Brucella* species demonstrated the presence of *Brucella* DNA in different wildlife hosts and further confirmed possible circulation of the zoonotic species of *Brucella* (*B*. *melitensis*) in buffaloes and giraffe. However, there were disparities between the serological and molecular findings concerning brucellosis in that not all animals positive for Brucella antibodies were positive by DNA analysis. These findings augment Alsubaie and co-workers’ [44] findings where serological diagnosis in human patients did not correlate with pathogen isolation by culture. This could be partly due to transient presence of *Brucella* antigens in blood where they are detected only for a short period around abortions, whereas antibodies persist for longer durations in the absence of antigens [45]. Caution should therefore be exercised in the choice of the diagnostic assay, based on the study objective(s). Nonetheless, the molecular findings of *Brucella melitensis* DNA in buffalo and giraffe further strengthen the evidence that wildlife harbors the pathogens and may have active infections that could be transmissible to susceptible hosts, especially humans, given that *B*. *melitensis*is a major cause of bloodstream infections and febrile illness in pastoral communities in eastern Africa [26,46].

This study utilized archived samples collected retrospectively over 10 years, which limited our ability to fully evaluate the contribution of different variables to the epidemiology of zoonotic diseases under investigation. Therefore, active nationwide surveillance with and systematic sampling approach could be conducted to fully explore the epidemiology of *Brucella*, *Coxiella* and RVFV in wildlife populations in Kenya, preferably augmented with laboratory surveillance.

## Supporting information

S1 Table Oligonucleotide primers and probes. (TIF)

S1 Fig Amplification plots for *B*. *abortus* and *B*. *melitensis*. (TIF)
