# Supplementary figures and images for: Evidence of co-exposure with Brucella spp, Coxiella burnetii, and Rift Valley fever virus among various species of wildlife in Kenya
Source: PLoS Negl Trop Dis. 2022 Aug 8;16(8):e0010596. doi: 10.1371/journal.pntd.0010596 (PMC9359551; doi:10.1371/journal.pntd.0010596)

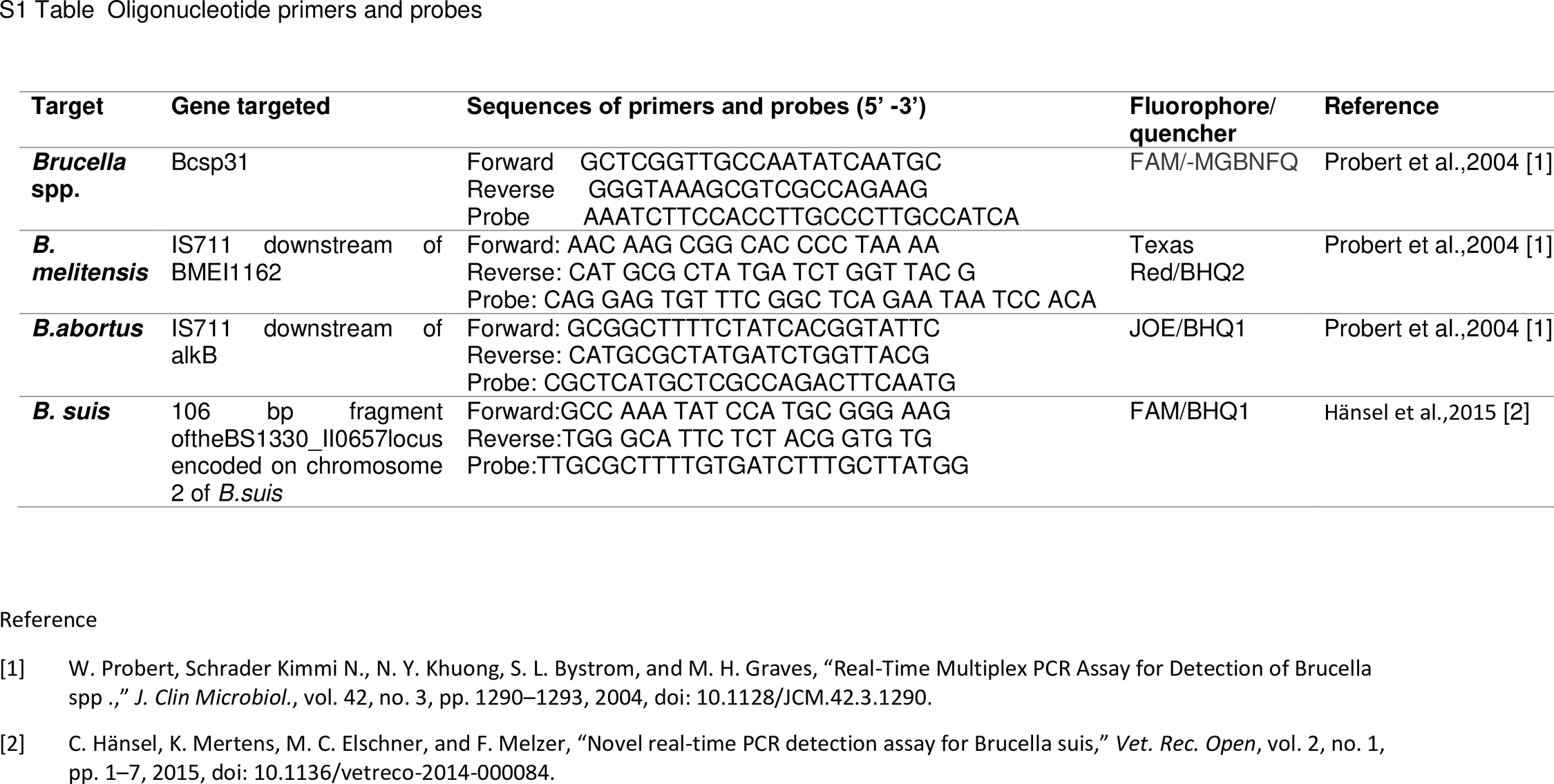

Supplement: S1 Table — (TIF) [file pntd.0010596.s001.tif]

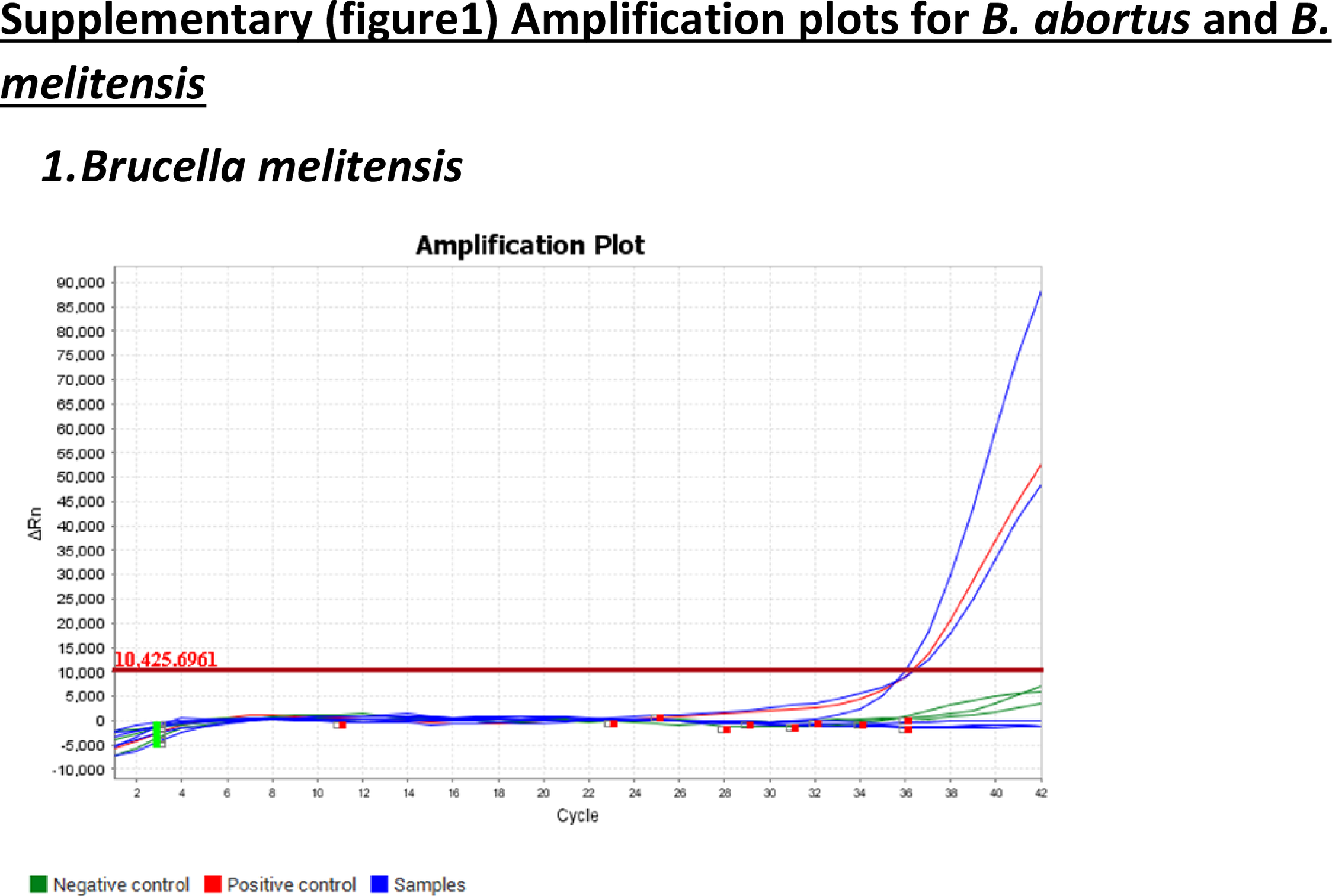

Supplement: S1 Fig — (TIF) [file pntd.0010596.s002.tif]
